# 2-Amino-4-(4-chloro­phen­yl)-7,7-dimethyl-5-oxo-5,6,7,8-tetra­hydro-4*H*-chromene-3-carbonitrile propan-2-one monosolvate

**DOI:** 10.1107/S1600536812024142

**Published:** 2012-05-31

**Authors:** Shaaban K. Mohamed, Mehmet Akkurt, Muhammad N. Tahir, Antar A. Abdelhamid, Mustafa R. Albayati

**Affiliations:** a Chemistry and Environmental Division, Manchester Metropolitan University, Manchester M1 5GD, England; b Department of Physics, Faculty of Sciences, Erciyes University, 38039 Kayseri, Turkey; c University of Sargodha, Department of Physics, Sargodha, Pakistan; d Kirkuk University, College of Science, Department of Chemistry, Kirkuk, Iraq

## Abstract

In the title compound, C_18_H_17_ClN_2_O_2_·C_3_H_6_O, the 4*H*-pyran ring is nearly planar [maximum deviation = −0.108 (1) Å] and the cyclo­hexene ring is puckered [puckering parameters *Q*_T_ = 0.4596 (17) Å, θ = 55.9 (2)° and ϕ = 226.5 (3)°]. The 4*H*-pyran ring is approximately perpendicular to the benzene ring [dihedral angle = 84.35 (7)°] and is almost coplanar with the mean plane of the cyclo­hexene ring [dihedral angle = 8.64 (7)°]. In the crystal, inversion-related main mol­ecules are linked into dimers by pairs of N—H⋯N hydrogen bonds, generating an *R*_2_^2^(12) graph-set motif. These dimers are further connected by N—H⋯O and C—H⋯N hydrogen bonds, forming a layer structure extending parallel to the (011) plane. In addition, the mol­ecules within the layers inter­act with each other *via* C—H⋯π inter­actions.

## Related literature

For the synthesis of chromene compounds, see: Coujon *et al.* (2002 ▶). For the bioactivity of chromene compounds see: Kaye & Nocanda (2002 ▶). For similar structures, see: Hu *et al.* (2012 ▶); Mohamed *et al.* (2012 ▶). For bond-length data, see: Allen *et al.* (1987 ▶). For puckering parameters, see: Cremer & Pople (1975 ▶). For hydrogen-bond motifs, see: Bernstein *et al.* (1995 ▶).
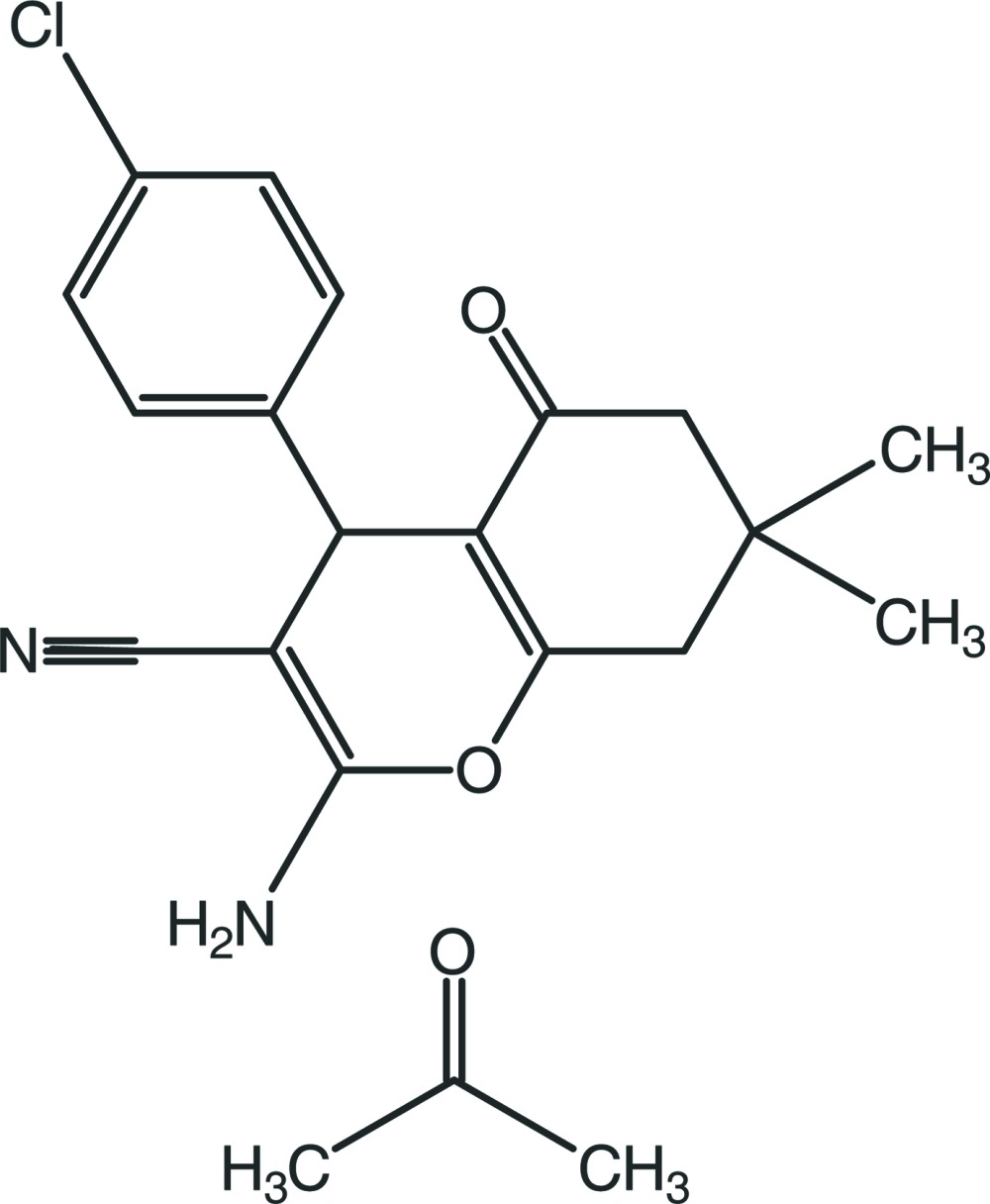


## Experimental

### 

#### Crystal data

C_18_H_17_ClN_2_O_2_·C_3_H_6_O
*M* *_r_* = 386.86
Triclinic, 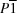

*a* = 8.1707 (2) Å
*b* = 9.4386 (2) Å
*c* = 13.5192 (4) Å
α = 84.446 (1)°
β = 82.546 (2)°
γ = 78.625 (1)°
*V* = 1010.76 (4) Å^3^
*Z* = 2
Mo *K*α radiation
μ = 0.21 mm^−1^
*T* = 296 K
0.28 × 0.25 × 0.23 mm

#### Data collection

Bruker Kappa APEXII CCD diffractometer
Absorption correction: multi-scan (*SADABS*; Bruker, 2005 ▶) *T*_min_ = 0.942, *T*_max_ = 0.952
16320 measured reflections
4769 independent reflections
3390 reflections with *I* > 2σ(*I*)
*R*_int_ = 0.027

#### Refinement

*R*[*F* ^2^ > 2σ(*F* ^2^)] = 0.045
*wR*(*F* ^2^) = 0.126
*S* = 1.04
4769 reflections
248 parameters
H-atom parameters constrained
Δρ_max_ = 0.33 e Å^−3^
Δρ_min_ = −0.34 e Å^−3^

### 

Data collection: *APEX2* (Bruker, 2007 ▶); cell refinement: *SAINT* (Bruker, 2007 ▶); data reduction: *SAINT*; program(s) used to solve structure: *SHELXS97* (Sheldrick, 2008 ▶); program(s) used to refine structure: *SHELXL97* (Sheldrick, 2008 ▶); molecular graphics: *ORTEP-3 for Windows* (Farrugia, 1997 ▶) and *PLATON* (Spek, 2009 ▶); software used to prepare material for publication: *WinGX* (Farrugia, 1999 ▶) and *PLATON*.

## Supplementary Material

Crystal structure: contains datablock(s) global, I. DOI: 10.1107/S1600536812024142/xu5550sup1.cif

Structure factors: contains datablock(s) I. DOI: 10.1107/S1600536812024142/xu5550Isup2.hkl

Supplementary material file. DOI: 10.1107/S1600536812024142/xu5550Isup3.cml

Additional supplementary materials:  crystallographic information; 3D view; checkCIF report

## Figures and Tables

**Table 1:** Hydrogen-bond geometry (Å, °) *Cg*1 and *Cg*2 are the centroids of the O1/C7–C11 and C1–C6 rings, respectively.

*D*—H⋯*A*	*D*—H	H⋯*A*	*D*⋯*A*	*D*—H⋯*A*
N2—H2*A*⋯N1^i^	0.86	2.30	3.1552 (19)	171
N2—H2*B*⋯O2^ii^	0.86	2.15	2.9949 (18)	167
C2—H2⋯N1^iii^	0.93	2.51	3.234 (2)	135
C17—H17*A*⋯*Cg*2^iv^	0.96	2.93	3.8221 (18)	155

Symmetry codes: (i) 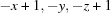; (ii) 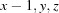; (iii) 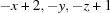; (iv) 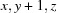.

**Fig. 1.**
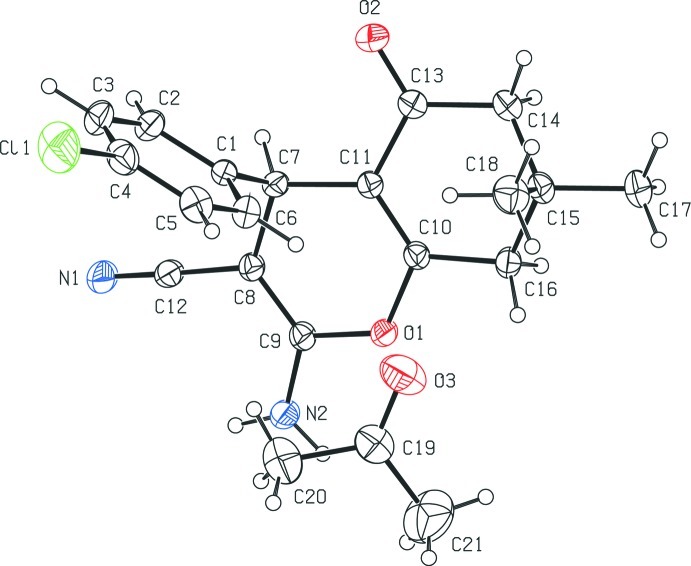
View of the title compound with the atom numbering scheme. Displacement ellipsoids for non-H atoms are drawn at the 30% probability level.

**Fig. 2.**
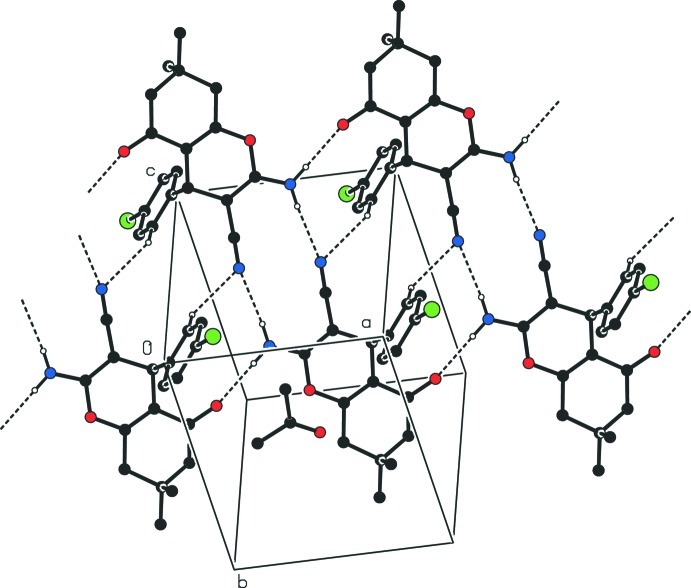
View of the dimers formed by pairs of N—H···N hydrogen bonds, with an R22(12) motif and the N—H···O and C—H···N hydrogen bonds which connect the dimers with each other. H atoms not involved in hydrogen bonds have been omitted for clarity.

## supplementary crystallographic information

### Comment

Chromene compounds are important group of oxygen heterocycles. They have employed as useful intermediate in the synthesis of a wide range of natural products (Coujon *et al.*, 2002). Such compounds have exhibited anti-depressant, anti-hypertensive as well as anti-ischaemic properties (Kaye & Nocanda, 2002). This triggered us to extend our on-going research program in synthesis of bioactive molecules and their pharmaceutical applications towards the synthesis of chromene nucleus containing compounds. We report in this study the synthesis and crystal structure study of 2-amino-4-(4-chlorophenyl)-7,7-dimethyl-5-oxo-5,6,7,8-tetrahydro-4*H*-chromene-3-carbonitrile–propan-2-one (1:1).

In the title compound (I), (Fig. 1), the C10/C11/C13–C16 cyclohexene ring is puckered with the puckering parameters (Cremer & Pople, 1975) of Q_T_ = 0.4596 (17) Å, θ = 55.9 (2)° and φ = 226.5 (3)°. The O1/C7–C11 4*H*-pyran ring is nearly planar with a maximum deviation of -0.108 (1) Å for C7 and is approximately perpendicular to the C1–C6 benzene ring [dihedral angle = 84.35 (7)°] and is almost co-planar with the mean plane of the cyclohexene ring [dihedral angle = 8.64 (7) °]. Bond lengths (Allen *et al.*, 1987) and angles of the title compound are within normal ranges and are comparable to similar structures (Hu *et al.*, 2012; Mohamed *et al.*, 2012).

In the crystal, a pair of intermolecular N—H···N hydrogen bonds link the main molecules into an inversion dimer, generating an *R*_2_^2^(12) graph-set motif (Bernstein *et al.*, 1995; Table 1, Fig. 2). The dimers are further connected by N—H···O and C—H···N hydrogen bonds, forming a layer of molecules parallel to (011) (Table 1, Fig. 2). The layers are interconnected by weak C—H···π interactions, producing a three-dimensional network.

### Experimental

A mixture of 140 mg (1 mmol) 5,5-dimethylcyclohexane-1,3-dione, 140 mg (1 mmol) 4-chlorobenzaldehyde and 123 mg (4-aminophenyl)methanol in 50 ml ethanol was refluxed for 5 h. The excess solvent was removed under vacuum and the residual resin was washed with cold acetone. The solid that formed was filtered off, washed with cold ethanol, well drained then recrystallized from a mixture of ethanol–acetone (1:1). Crystals obtained were in good quality and suitable for X-ray diffraction (m.p. 461 K).

### Refinement

H atoms were positioned geometrically and refined by using a riding model, with N—H = 0.86 Å and C—H = 0.93 Å (aromatic), 0.96 Å (methyl), 0.97 Å (methylene) and 0.98 Å (methine), with *U*_iso_(H) = 1.5*U*_eq_(C) for methyl groups and *U*_iso_(H) = 1.2*U*_eq_(C,N) for others.

### Crystal data

**Table:**

C_18_H_17_ClN_2_O_2_·C_3_H_6_O	*Z* = 2
*M**_r_* = 386.86	*F*(000) = 408
Triclinic, *P*1	*D*_x_ = 1.271 Mg m^−^^3^
Hall symbol: -P 1	Mo *K*α radiation, λ = 0.71073 Å
*a* = 8.1707 (2) Å	Cell parameters from 430 reflections
*b* = 9.4386 (2) Å	θ = 2.2–21°
*c* = 13.5192 (4) Å	µ = 0.21 mm^−^^1^
α = 84.446 (1)°	*T* = 296 K
β = 82.546 (2)°	Prism, colourless
γ = 78.625 (1)°	0.28 × 0.25 × 0.23 mm
*V* = 1010.76 (4) Å^3^	

### Data collection

**Table:**

Bruker Kappa APEXII CCD diffractometer	4769 independent reflections
Radiation source: fine-focus sealed tube	3390 reflections with *I* > 2σ(*I*)
Graphite monochromator	*R*_int_ = 0.027
Detector resolution: 0.81 pixels mm^-1^	θ_max_ = 27.9°, θ_min_ = 1.5°
ω scans	*h* = −9→10
Absorption correction: multi-scan (*SADABS*; Bruker, 2005)	*k* = −12→12
*T*_min_ = 0.942, *T*_max_ = 0.952	*l* = −17→17
16320 measured reflections	

### Refinement

**Table:**

Refinement on *F*^2^	Primary atom site location: structure-invariant direct methods
Least-squares matrix: full	Secondary atom site location: difference Fourier map
*R*[*F*^2^ > 2σ(*F*^2^)] = 0.045	Hydrogen site location: inferred from neighbouring sites
*wR*(*F*^2^) = 0.126	H-atom parameters constrained
*S* = 1.04	*w* = 1/[σ^2^(*F*_o_^2^) + (0.0552*P*)^2^ + 0.2106*P*] where *P* = (*F*_o_^2^ + 2*F*_c_^2^)/3
4769 reflections	(Δ/σ)_max_ < 0.001
248 parameters	Δρ_max_ = 0.33 e Å^−^^3^
0 restraints	Δρ_min_ = −0.34 e Å^−^^3^

### Special details

**Table:**

Geometry. Bond distances, angles *etc*. have been calculated using the rounded fractional coordinates. All su's are estimated from the variances of the (full) variance-covariance matrix. The cell e.s.d.'s are taken into account in the estimation of distances, angles and torsion angles
Refinement. Refinement on *F*^2^ for ALL reflections except those flagged by the user for potential systematic errors. Weighted *R*-factors *wR* and all goodnesses of fit *S* are based on *F*^2^, conventional *R*-factors *R* are based on *F*, with *F* set to zero for negative *F*^2^. The observed criterion of *F*^2^ > σ(*F*^2^) is used only for calculating -*R*-factor-obs *etc*. and is not relevant to the choice of reflections for refinement. *R*-factors based on *F*^2^ are statistically about twice as large as those based on *F*, and *R*-factors based on ALL data will be even larger.

### Fractional atomic coordinates and isotropic or equivalent isotropic displacement parameters (Å^2^)

**Table:**

	*x*	*y*	*z*	*U*_iso_*/*U*_eq_	
Cl1	1.23217 (8)	−0.04097 (7)	0.07282 (5)	0.0908 (3)	
O1	0.49042 (12)	0.48205 (10)	0.35120 (8)	0.0400 (3)	
O2	1.05221 (14)	0.51811 (13)	0.37189 (11)	0.0565 (5)	
N1	0.67987 (18)	0.01830 (15)	0.51140 (12)	0.0536 (5)	
N2	0.36160 (16)	0.29958 (14)	0.40904 (11)	0.0480 (5)	
C1	0.92881 (17)	0.24000 (15)	0.30888 (11)	0.0342 (4)	
C2	1.05201 (19)	0.12327 (17)	0.33381 (14)	0.0460 (5)	
C3	1.1449 (2)	0.03660 (19)	0.26177 (16)	0.0555 (6)	
C4	1.1148 (2)	0.06724 (19)	0.16419 (15)	0.0531 (6)	
C5	0.9934 (2)	0.18223 (19)	0.13653 (14)	0.0513 (6)	
C6	0.9008 (2)	0.26753 (17)	0.20960 (12)	0.0430 (5)	
C7	0.82134 (17)	0.32702 (15)	0.39096 (11)	0.0337 (4)	
C8	0.65888 (18)	0.27155 (15)	0.42120 (11)	0.0335 (4)	
C9	0.50856 (18)	0.34425 (15)	0.39607 (11)	0.0342 (4)	
C10	0.62537 (18)	0.55179 (15)	0.33926 (11)	0.0334 (4)	
C11	0.77873 (17)	0.48648 (15)	0.36014 (11)	0.0326 (4)	
C12	0.66848 (18)	0.13136 (16)	0.47074 (12)	0.0380 (4)	
C13	0.90997 (19)	0.57350 (16)	0.35344 (12)	0.0388 (5)	
C14	0.8623 (2)	0.73361 (17)	0.32758 (14)	0.0463 (5)	
C15	0.7272 (2)	0.77363 (16)	0.25591 (12)	0.0409 (5)	
C16	0.57651 (18)	0.70488 (15)	0.30076 (12)	0.0387 (5)	
C17	0.6697 (2)	0.93797 (18)	0.24419 (17)	0.0592 (7)	
C18	0.7976 (2)	0.7186 (2)	0.15339 (14)	0.0564 (6)	
O3	0.5451 (2)	0.50051 (18)	0.11608 (13)	0.0843 (7)	
C19	0.4295 (3)	0.4388 (2)	0.11692 (15)	0.0603 (7)	
C20	0.4577 (4)	0.2802 (3)	0.1159 (2)	0.0995 (13)	
C21	0.2546 (3)	0.5175 (3)	0.1176 (3)	0.1166 (13)	
H2	1.07260	0.10280	0.40020	0.0550*	
H2A	0.35510	0.21450	0.43660	0.0580*	
H2B	0.27340	0.35580	0.38990	0.0580*	
H3	1.22690	−0.04170	0.27950	0.0670*	
H5	0.97400	0.20230	0.06990	0.0620*	
H6	0.81800	0.34500	0.19150	0.0520*	
H7	0.88370	0.31470	0.44940	0.0410*	
H14A	0.96180	0.76980	0.29740	0.0560*	
H14B	0.82190	0.78160	0.38870	0.0560*	
H16A	0.51530	0.76070	0.35500	0.0460*	
H16B	0.50160	0.70960	0.25000	0.0460*	
H17A	0.76440	0.98270	0.22010	0.0890*	
H17B	0.62060	0.97280	0.30780	0.0890*	
H17C	0.58780	0.96160	0.19740	0.0890*	
H18A	0.71240	0.74390	0.10870	0.0850*	
H18B	0.83210	0.61510	0.16000	0.0850*	
H18C	0.89240	0.76210	0.12710	0.0850*	
H20A	0.41990	0.25490	0.05680	0.1490*	
H20B	0.39630	0.24090	0.17400	0.1490*	
H20C	0.57550	0.24120	0.11620	0.1490*	
H21A	0.25020	0.61800	0.12620	0.1750*	
H21B	0.18550	0.47730	0.17170	0.1750*	
H21C	0.21410	0.50840	0.05540	0.1750*	

### Atomic displacement parameters (Å^2^)

**Table:**

	*U*^11^	*U*^22^	*U*^33^	*U*^12^	*U*^13^	*U*^23^
Cl1	0.0881 (4)	0.0749 (4)	0.1017 (5)	−0.0010 (3)	0.0216 (3)	−0.0411 (3)
O1	0.0293 (5)	0.0326 (5)	0.0566 (7)	−0.0079 (4)	−0.0069 (5)	0.0111 (5)
O2	0.0351 (6)	0.0490 (7)	0.0871 (10)	−0.0102 (5)	−0.0169 (6)	0.0050 (6)
N1	0.0469 (8)	0.0429 (8)	0.0700 (10)	−0.0113 (6)	−0.0163 (7)	0.0197 (7)
N2	0.0314 (7)	0.0391 (7)	0.0715 (10)	−0.0099 (6)	−0.0074 (6)	0.0151 (7)
C1	0.0271 (7)	0.0287 (7)	0.0468 (9)	−0.0067 (6)	−0.0050 (6)	0.0019 (6)
C2	0.0354 (8)	0.0405 (9)	0.0591 (11)	−0.0009 (7)	−0.0090 (7)	0.0036 (8)
C3	0.0384 (9)	0.0392 (9)	0.0830 (14)	0.0046 (7)	−0.0040 (9)	−0.0041 (9)
C4	0.0451 (10)	0.0430 (9)	0.0698 (13)	−0.0097 (8)	0.0099 (8)	−0.0169 (8)
C5	0.0579 (11)	0.0464 (10)	0.0500 (10)	−0.0122 (8)	−0.0019 (8)	−0.0055 (8)
C6	0.0431 (9)	0.0350 (8)	0.0497 (10)	−0.0035 (7)	−0.0075 (7)	−0.0016 (7)
C7	0.0304 (7)	0.0318 (7)	0.0395 (8)	−0.0065 (6)	−0.0093 (6)	0.0036 (6)
C8	0.0330 (7)	0.0294 (7)	0.0371 (8)	−0.0063 (6)	−0.0038 (6)	0.0036 (6)
C9	0.0344 (7)	0.0308 (7)	0.0365 (8)	−0.0083 (6)	−0.0020 (6)	0.0037 (6)
C10	0.0323 (7)	0.0291 (7)	0.0384 (8)	−0.0078 (6)	−0.0012 (6)	−0.0006 (6)
C11	0.0306 (7)	0.0287 (7)	0.0383 (8)	−0.0060 (6)	−0.0031 (6)	−0.0011 (6)
C12	0.0315 (7)	0.0387 (8)	0.0430 (8)	−0.0066 (6)	−0.0072 (6)	0.0050 (7)
C13	0.0363 (8)	0.0367 (8)	0.0443 (9)	−0.0093 (6)	−0.0050 (6)	−0.0024 (6)
C14	0.0442 (9)	0.0355 (8)	0.0619 (11)	−0.0141 (7)	−0.0079 (8)	−0.0013 (7)
C15	0.0423 (8)	0.0298 (8)	0.0508 (9)	−0.0108 (6)	−0.0030 (7)	0.0019 (7)
C16	0.0360 (8)	0.0286 (7)	0.0488 (9)	−0.0032 (6)	−0.0022 (7)	0.0016 (6)
C17	0.0613 (11)	0.0318 (9)	0.0848 (14)	−0.0125 (8)	−0.0127 (10)	0.0085 (9)
C18	0.0596 (11)	0.0564 (11)	0.0512 (11)	−0.0167 (9)	0.0037 (8)	0.0048 (8)
O3	0.0928 (12)	0.0868 (11)	0.0863 (12)	−0.0419 (10)	−0.0209 (9)	−0.0046 (9)
C19	0.0682 (13)	0.0644 (12)	0.0506 (11)	−0.0211 (10)	−0.0033 (9)	−0.0024 (9)
C20	0.109 (2)	0.0637 (15)	0.132 (3)	−0.0259 (15)	−0.0305 (18)	0.0047 (15)
C21	0.0753 (18)	0.115 (2)	0.140 (3)	0.0068 (16)	0.0197 (17)	−0.006 (2)

### Geometric parameters (Å, º)

**Table:**

Cl1—C4	1.743 (2)	C15—C17	1.528 (2)
O1—C9	1.3697 (17)	C15—C18	1.529 (2)
O1—C10	1.3765 (18)	C2—H2	0.9300
O2—C13	1.223 (2)	C3—H3	0.9300
O3—C19	1.202 (3)	C5—H5	0.9300
N1—C12	1.145 (2)	C6—H6	0.9300
N2—C9	1.335 (2)	C7—H7	0.9800
N2—H2B	0.8600	C14—H14B	0.9700
N2—H2A	0.8600	C14—H14A	0.9700
C1—C2	1.385 (2)	C16—H16A	0.9700
C1—C6	1.382 (2)	C16—H16B	0.9700
C1—C7	1.522 (2)	C17—H17B	0.9600
C2—C3	1.383 (3)	C17—H17C	0.9600
C3—C4	1.366 (3)	C17—H17A	0.9600
C4—C5	1.377 (2)	C18—H18B	0.9600
C5—C6	1.384 (2)	C18—H18C	0.9600
C7—C11	1.507 (2)	C18—H18A	0.9600
C7—C8	1.515 (2)	C19—C20	1.470 (3)
C8—C9	1.351 (2)	C19—C21	1.474 (4)
C8—C12	1.417 (2)	C20—H20A	0.9600
C10—C11	1.335 (2)	C20—H20B	0.9600
C10—C16	1.483 (2)	C20—H20C	0.9600
C11—C13	1.463 (2)	C21—H21A	0.9600
C13—C14	1.503 (2)	C21—H21B	0.9600
C14—C15	1.530 (2)	C21—H21C	0.9600
C15—C16	1.533 (2)		
			
C9—O1—C10	118.92 (11)	C4—C5—H5	121.00
C9—N2—H2B	120.00	C6—C5—H5	121.00
H2A—N2—H2B	120.00	C1—C6—H6	119.00
C9—N2—H2A	120.00	C5—C6—H6	119.00
C6—C1—C7	122.04 (13)	C1—C7—H7	108.00
C2—C1—C6	118.08 (14)	C8—C7—H7	108.00
C2—C1—C7	119.74 (14)	C11—C7—H7	108.00
C1—C2—C3	121.22 (17)	C13—C14—H14A	109.00
C2—C3—C4	119.25 (16)	C13—C14—H14B	109.00
Cl1—C4—C3	119.52 (14)	C15—C14—H14A	109.00
C3—C4—C5	121.21 (17)	C15—C14—H14B	109.00
Cl1—C4—C5	119.27 (15)	H14A—C14—H14B	108.00
C4—C5—C6	118.86 (17)	C10—C16—H16A	109.00
C1—C6—C5	121.37 (15)	C10—C16—H16B	109.00
C1—C7—C11	112.80 (12)	C15—C16—H16A	109.00
C1—C7—C8	110.40 (12)	C15—C16—H16B	109.00
C8—C7—C11	108.40 (12)	H16A—C16—H16B	108.00
C7—C8—C9	123.08 (13)	C15—C17—H17A	109.00
C7—C8—C12	117.87 (13)	C15—C17—H17B	109.00
C9—C8—C12	118.83 (14)	C15—C17—H17C	109.00
N2—C9—C8	128.24 (14)	H17A—C17—H17B	109.00
O1—C9—N2	110.31 (12)	H17A—C17—H17C	110.00
O1—C9—C8	121.45 (13)	H17B—C17—H17C	109.00
O1—C10—C11	122.99 (13)	C15—C18—H18A	109.00
O1—C10—C16	111.12 (12)	C15—C18—H18B	109.00
C11—C10—C16	125.89 (14)	C15—C18—H18C	109.00
C10—C11—C13	118.65 (13)	H18A—C18—H18B	109.00
C7—C11—C10	122.53 (13)	H18A—C18—H18C	109.00
C7—C11—C13	118.81 (12)	H18B—C18—H18C	110.00
N1—C12—C8	178.38 (17)	O3—C19—C20	120.9 (2)
O2—C13—C11	120.84 (14)	O3—C19—C21	122.05 (19)
O2—C13—C14	121.28 (14)	C20—C19—C21	117.0 (2)
C11—C13—C14	117.84 (13)	C19—C20—H20A	109.00
C13—C14—C15	113.74 (13)	C19—C20—H20B	110.00
C16—C15—C17	108.70 (13)	C19—C20—H20C	109.00
C16—C15—C18	110.90 (13)	H20A—C20—H20B	109.00
C17—C15—C18	108.98 (15)	H20A—C20—H20C	109.00
C14—C15—C18	109.79 (14)	H20B—C20—H20C	109.00
C14—C15—C16	108.05 (13)	C19—C21—H21A	109.00
C14—C15—C17	110.41 (13)	C19—C21—H21B	109.00
C10—C16—C15	113.11 (12)	C19—C21—H21C	109.00
C1—C2—H2	119.00	H21A—C21—H21B	109.00
C3—C2—H2	119.00	H21A—C21—H21C	110.00
C2—C3—H3	120.00	H21B—C21—H21C	109.00
C4—C3—H3	120.00		
			
C9—O1—C10—C16	−173.19 (12)	C8—C7—C11—C10	−15.79 (19)
C10—O1—C9—N2	174.51 (12)	C8—C7—C11—C13	163.79 (13)
C10—O1—C9—C8	−5.5 (2)	C7—C8—C9—N2	172.17 (15)
C9—O1—C10—C11	7.0 (2)	C12—C8—C9—O1	177.72 (13)
C7—C1—C2—C3	176.02 (14)	C12—C8—C9—N2	−2.3 (2)
C2—C1—C6—C5	−0.5 (2)	C7—C8—C9—O1	−7.8 (2)
C7—C1—C6—C5	−176.29 (15)	O1—C10—C11—C7	5.0 (2)
C6—C1—C2—C3	0.1 (2)	O1—C10—C11—C13	−174.58 (13)
C2—C1—C7—C8	−94.69 (16)	C16—C10—C11—C7	−174.80 (14)
C2—C1—C7—C11	143.88 (14)	C16—C10—C11—C13	5.6 (2)
C6—C1—C7—C8	81.04 (17)	O1—C10—C16—C15	−162.22 (12)
C6—C1—C7—C11	−40.39 (19)	C11—C10—C16—C15	17.6 (2)
C1—C2—C3—C4	0.3 (3)	C7—C11—C13—O2	0.4 (2)
C2—C3—C4—Cl1	179.66 (13)	C7—C11—C13—C14	−176.99 (14)
C2—C3—C4—C5	−0.3 (3)	C10—C11—C13—O2	−180.00 (17)
Cl1—C4—C5—C6	180.00 (15)	C10—C11—C13—C14	2.6 (2)
C3—C4—C5—C6	−0.1 (3)	O2—C13—C14—C15	148.89 (16)
C4—C5—C6—C1	0.5 (3)	C11—C13—C14—C15	−33.7 (2)
C1—C7—C8—C12	67.77 (17)	C13—C14—C15—C16	53.89 (18)
C11—C7—C8—C9	17.28 (19)	C13—C14—C15—C17	172.65 (15)
C11—C7—C8—C12	−168.22 (13)	C13—C14—C15—C18	−67.18 (18)
C1—C7—C11—C10	106.77 (16)	C14—C15—C16—C10	−45.43 (17)
C1—C7—C8—C9	−106.73 (16)	C17—C15—C16—C10	−165.27 (14)
C1—C7—C11—C13	−73.65 (17)	C18—C15—C16—C10	74.94 (17)

### Hydrogen-bond geometry (Å, º)

Cg1 and Cg2 are the centroids of the O1/C7–C11 and C1–C6 rings, respectively.

**Table:**

*D*—H···*A*	*D*—H	H···*A*	*D*···*A*	*D*—H···*A*
N2—H2*A*···N1^i^	0.86	2.30	3.1552 (19)	171
N2—H2*B*···O2^ii^	0.86	2.15	2.9949 (18)	167
C2—H2···N1^iii^	0.93	2.51	3.234 (2)	135
C6—H6···*Cg*1	0.93	2.76	3.0785 (17)	101
C17—H17*A*···*Cg*2^iv^	0.96	2.93	3.8221 (18)	155

Symmetry codes: (i) −*x*+1, −*y*, −*z*+1; (ii) *x*−1, *y*, *z*; (iii) −*x*+2, −*y*, −*z*+1; (iv) *x*, *y*+1, *z*.
